# Brood indicators are an early warning signal of honey bee colony loss—a simulation-based study

**DOI:** 10.1371/journal.pone.0302907

**Published:** 2024-05-16

**Authors:** Jürgen Groeneveld, Richard Odemer, Fabrice Requier

**Affiliations:** 1 Department of Ecological Modelling, Helmholtz Centre for Environmental Research–UFZ, Leipzig, Germany; 2 Institute for Bee Protection, Julius Kühn-Institut (JKI)–Federal Research Centre for Cultivated Plants, Braunschweig, Germany; 3 Université Paris-Saclay, CNRS, IRD, UMR Évolution, Génomes, Comportement et Écologie, 91198, Gif-sur-Yvette, France; University of Carthage, TUNISIA

## Abstract

Honey bees (*Apis mellifera*) are exposed to multiple stressors such as pesticides, lack of forage, and diseases. It is therefore a long-standing aim to develop robust and meaningful indicators of bee vitality to assist beekeepers While established indicators often focus on expected colony winter mortality based on adult bee abundance and honey reserves at the beginning of the winter, it would be useful to have indicators that allow detection of stress effects earlier in the year to allow for adaptive management. We used the established honey bee simulation model BEEHAVE to explore the potential of different indicators such as population size, number of capped brood cells, flight activity, abundance of Varroa mites, honey stores and a brood-bee ratio. We implemented two types of stressors in our simulations: 1) parasite pressure, i.e. sub-optimal Varroa treatment by the beekeeper (hereafter referred as Biotic stress) and 2) temporal forage gaps in spring and autumn (hereafter referred as Environmental stress). Neither stressor type could be detected by bee abundance or honey reserves at the end of the first year. However, all response variables used in this study did reveal early warning signals during the course of the year. The most reliable and useful measures seem to be related to brood and the abundance of Varroa mites at the end of the year. However, while in the model we have full access to time series of variables from stressed and unstressed colonies, knowledge of these variables in the field is challenging. We discuss how our findings can nevertheless be used to develop practical early warning indicators. As a next step in the interactive development of such indicators we suggest empirical studies on the importance of the number of capped brood cells at certain times of the year on bee population vitality.

## Introduction

Honey bees (*Apis mellifera*) together with other animal pollinators are vital for human well-being [1]. Honey bees make an important contribution to crop pollination [2] and wild plant reproduction [3]. However, honey bee colonies partly suffered increasing mortality rates since several decades worldwide [4–7]. In Europe, colony loss surveys showed an average colony mortality rate of 16.4% in winter 2017/2018 and 18.1% in winter 2019/2020 [4,5]. In Germany, winter mortality has remained relatively moderate at 12.5% over the past 10 years, with no clear trend over time [8]. This contrasts the US, where annual colony losses can account for up to half of beekeepers’ livestock [7].

The invasive parasitic mite *Varroa destructor* is recognized as a significant risk factor for honey bees, and plays a critical role as a vector for diseases such as deformed wing virus and acute bee paralysis virus. This mite-induced transmission is particularly important for winter losses as it can lead to colony collapse [8]. An important demonstration of this was made on the isolated island of Gotland. Honey bee colonies there were systematically infested with Varroa mites and left untreated. After three winters, only 17 of the original 150 colonies survived [9]. Other biotic risks are invasive predator species such as the Asian Hornet [10]. Honey bees also depend on a continuous supply of floral resources. Thus, land use change and the reduction of semi-natural areas in farmlands pose an additional risk to honey bees by creating foraging gaps [11–13]. Foraging gaps are suggested to occur in intensive farmland habitats where food supply is limited during the period when mass flowering crops (e.g. rapeseed and sunflower) are not flowering [14]. In addition to foraging gaps and diseases, pesticides [15], climate [16] and mismanagement [17,18] are emerging as potential stressors for honey bees. It is highly likely that these single stressors act together in a non-additive manner, i.e., the combined effect cannot be predicted by studying the stressors in isolation [19]. The presence of these multiple risk factors makes it difficult to identify rapid and standardized techniques to address colony losses. However, the adoption of observable indicators of bee health for adaptive management by beekeepers appears to be a practical and feasible perspective [20].

In a review, Requier [20] identified four main classes of bee health indicators by summarizing an EFSA (European Food Safety Authority) report [21]: 1) Queen presence and performance, 2) colony dynamics, 3) in-hive products (e.g. amount of pollen and bee bread and honey), and 4) antagonists (e.g. Varroa mites). A major requirement for these indicators to be practical is that they can be easily measured in the field by beekeepers. Colony dynamic indicators such as colony size, number of capped brood cells or brood pattern [22] are generally more relevant in this way than indicators measured at the individual level (which is more complex to observe). Colony level indicators are also more comprehensive because the bee colony is a superorganism that can compensate for stress experienced by individual workers [20,23].

Indicators that focus on individual assessments involving behavioural and physiological parameters of bees–such as measuring lipid stores [24], examining the "bacterial gut community" [25], or assessing flight activity [26]–are primarily investigated through experimental studies, as highlighted in the EFSA report [21]. It is important to note that the assessment of these individual indicators often requires the expertise of professionals using specialized equipment and analytical methods that are not usually available to beekeepers [26]. However, it is not clear which of the introduced indicators are the most suitable for tracking and anticipating colony loss.

To address this gap, mechanistic simulation models can be used as virtual laboratories [27,28] to systematically test and evaluate the utility of honey bee colony loss indicators [29]. Despite the acceptance of this workflow, to our knowledge there are no examples yet. In this study, we therefore aimed to use the established honey bee simulation model BEEHAVE [30] to assess the performance of a core of individual and colony traits as potential early warning signals of colony loss. The BEEHAVE model simulates a honey bee colony in a resource landscape. The model incorporates the temporal population dynamics of eggs, larvae, pupae, in-hive worker bees, and foraging bees. In addition, it takes into account the foraging process and the population dynamics of the Varroa mite (see section “The BEEHAVE model” and [30] for more details). Our focus was on colonies stressed by two major risk factors: early and late-season food shortages (Environmental stress) and the absence of Varroa treatment (Biotic stress). To identify useful indicators, we present the following analytic steps: 1) Effect of stress on the honey bee population persistence, 2) Correlations between different simulation responses, 3) Analysis of six potential indicators, 4) Testing the robustness of our results in respect to implementation details.

## Methods

### The BEEHAVE model

We used the BEEHAVE model (the “forbeemapp” updated version https://beehave-model.net/) to simulate the colony dynamic of honey bees. BEEHAVE is implemented in NetLogo [31] and we have used R [32] and the packages corrplot [33] and recolorize [34] for visualisations and analysis. BEEHAVE simulates a single honey bee colony. It includes three modules, which represent in-hive dynamics, Varroa mite dynamics and the corresponding transmission of the deformed wing virus, and foraging of nectar and pollen in the surrounding landscape. The landscape is characterized by its “flower patches”, i.e., arable fields, which are described by their area, distance to the hive, crop type and phenology, and nectar and pollen provided by the crop plant. Weather data determine the number of foraging hours per day. Recruitment of foragers to new flower patches via the bee’s waggle dance is also represented. Beekeeping practices such as Varroa treatment, honey harvesting, and swarm control are implemented and optional. An overview of the concepts and basic principles underlying the design of the model, following the standard ODD (Overview, Design, and Details [35,36]) can be found in the supplement of Becher et al. [30], while detailed documentation is available on the model’s website (https://beehave-model.net/). The modified BEEHAVE model version used for this study, input files, simulated data and R scripts to produce the figures are available at ZENODO (https://doi.org/10.5281/zenodo.10999499).

### Simulation experiments

**Fig 1:**
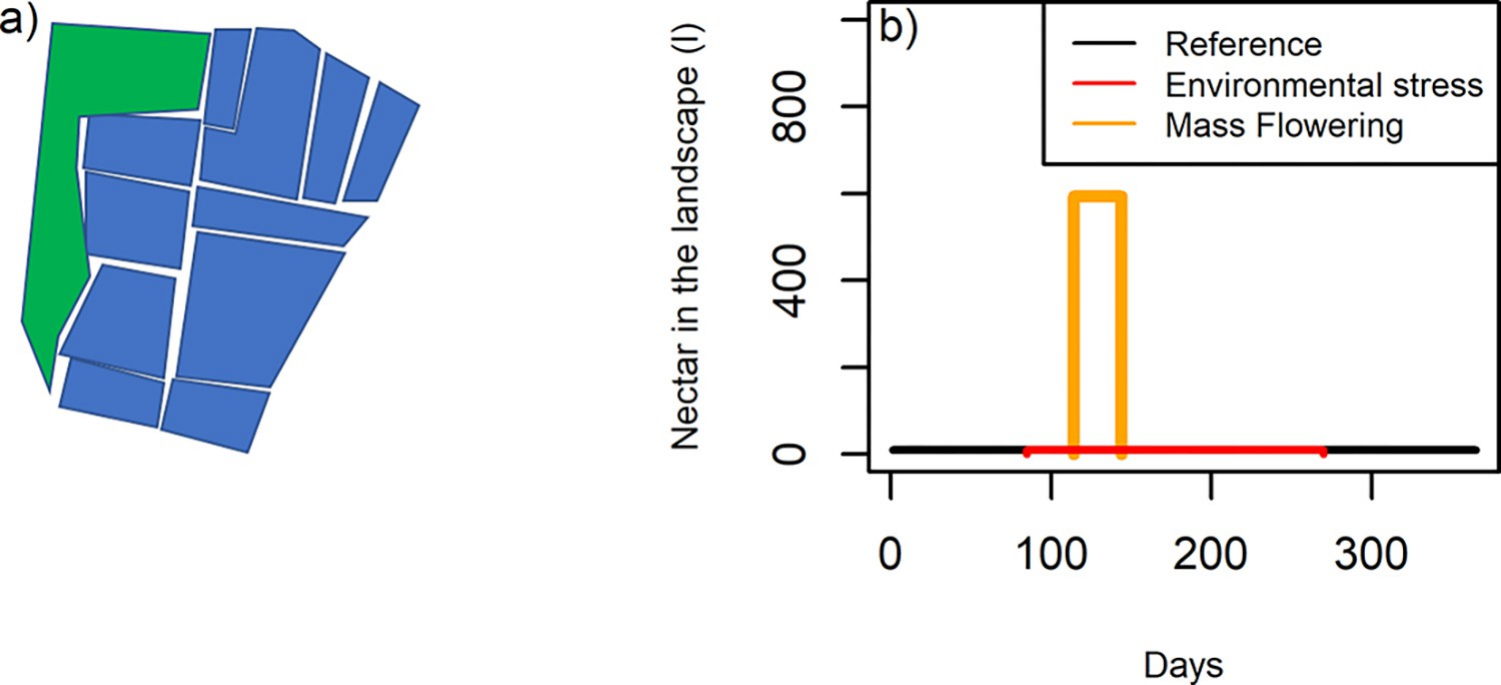
a) Stylized landscape (green patch seminatural grassland and blue patches oil seed rape). The total area of the patches is 79 hectares, the beehive is located in the centre of the landscape. b) This stylized landscape would translate into a short period of mass flowering of the oilseed rape and an assumed background resource available around the year from the seminatural grass land (black line) or during a reduced duration (Environmental stress scenario, red line). The amount of pollen which is not shown here shows the same temporal dynamics.

We simulated the development of a single beehive without stress and contrasted this scenario with two types of stressors (Environmental and Biotic stress) to investigate which of six indicators (1) number of adult bees, (2) number of capped brood cells (identical to number of pupae in the BEEHAVE model), (3) honey yield index (cumulated daily differences in hive weight, which is a good proxy for nectar yield and is used in monitoring campaigns such as Trachtnet (https://www.bienenkunde.rlp.de/Bienenkunde/Trachtnet/Waagenstandorte-Karte), (4) flight activity, (5) number of Varroa mites, and (6) brood-bee ratio, which is the sum of all eggs, larvae and pupae divided by the number of adult bees, may best serve as an early warning signal. The resource landscape around the beehive was oriented towards an agricultural landscape in Almke (52.34 N, 10.86 E) in northern Germany near the city of Wolfsburg. This study site was chosen because it happens to be the most homogeneous agricultural land use regime within a network of study sites in a larger research project (VIBee–Establishing digital indicators of bee vitality in agricultural landscapes). We simplified the resource landscape into 11 patches where oilseed rape was grown and one polygon that represented seminatural grassland (Fig 1A). The simulation started on the first of January with 10,000 foraging bees (in BEEHAVE overwintering bees are represented by foraging bees). The daily weather was based on the built-in data set of the Berlin weather from 2000 to 2006.

While the oilseed rape fields provided mass flowering resources for a short period of time (30 days, Fig 1B) the semi natural grassland provided a low amount of pollen and nectar throughout the year in the default setting. Based on field data [14], oilseed rape was assumed to flower for 30 days from day 114 (end of April) to day 144 (end of May). In the model, oilseed rape provides 0.349 g/m^2^ of pollen and 0.001 l/m^2^ of nectar (with a sugar concentration of 1.7 mol/l) during flowering ([37], Table 3.3). Seminatural grassland provides 0.006 g/m^2^ pollen and 0.00005 l/m^2^ nectar (with a sugar concentration of 1.08 mol/l) throughout the year. The values for the semi natural grassland were conservative estimates of background reserve availability based on data compiled by Horn [37].

**Fig 2:**
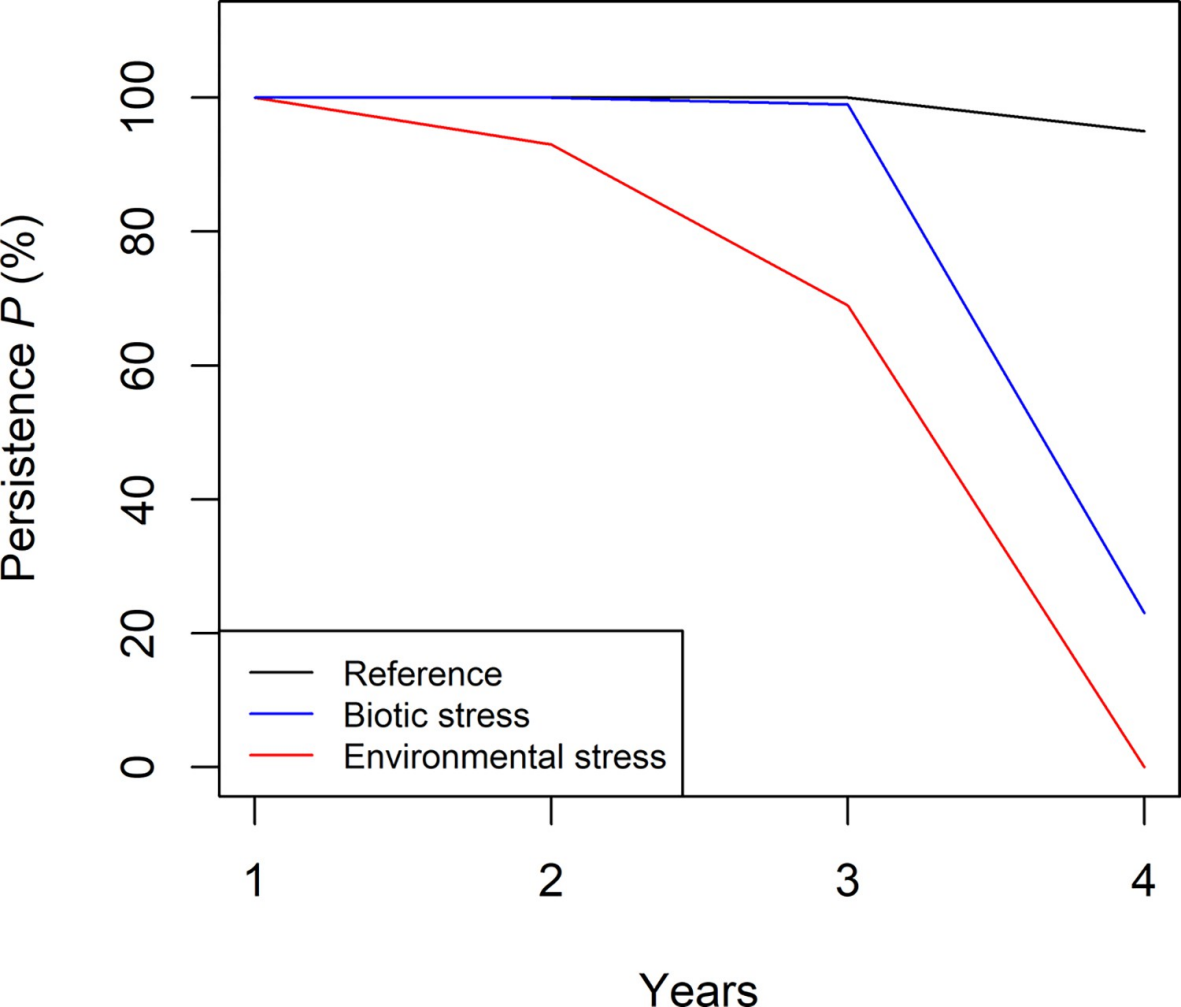
Persistence *P* measured as the percentage of honey bee colonies (n = 100) that had more than 5000 adult bees at the end of a simulated years for three scenarios.

In all simulations (except the "Biotic stress" scenario) the Varroa treatment was applied, honey was harvested and swarming was prevented. Therefore, we used the default parameter sets: remaining honey after harvest 10 kg, harvest threshold = 20 kg, i.e. when this threshold is reached honey will be harvested, harvest day 135, i.e. honey harvest is possible from this day on, harvesting period 85 days, i.e. this is the period starting at harvesting day where honey harvesting is allowed. We applied Varroa treatment as it was implemented in BEEHAVE, i.e., starting on day 270, phoretic mites experienced an additional 4% daily mortality for 40 days, which represented the application of an acaricide. We use the term phoretic here to describe mites that are attached to adult bees, as contrasted with mites that are in brood cells. In addition, all mites in brood cells were killed by the application of the acaricide (for a new Varroa control module for BEEHAVE that represents treatment using organic acids only, see [38]). Furthermore, all drone brood cells were removed several times (days: 100, 140, 180, 220, 240) to eliminate mites. Drone brood removal is considered an effective measure, since mites prefer to reproduce in drone cells [39] which makes it part of integrated pest management in beekeeping [40]. For the Environmental stress scenario, we reduced the temporal resource availability for the seminatural grassland from year-round to a period starting at day 85 and ending at day 270. The duration of limited resource availability was chosen so that honey bees were stressed but did not die in the first year, while colony survival starts to decrease after two years (Fig 2). In the Biotic stress scenario, we introduced stress by modelling mismanagement, i.e., no acaricide treatment was performed against the mite while drone brood removal was still carried out.

For each scenario, i.e. I) Reference, II) Environmental stress, III) Biotic stress, we ran 100 simulations that lasted 8 years. In case the colony died during this time, the simulation was continued with 0 bees to obtain time series of equal length. To avoid impacts of initial conditions all simulations ran for two years without stress. These first two years are considered and referred to as burn in phase, and dynamics during this phase are not further reported.

### Stress detection methods–identifying early warning signals

#### Population persistence

First, we measured persistence of the bee hives by observing the number of adult bees at the last day the of the year to investigate the impact of stress on the bee population. We reported persistence (Fig 2) as the proportion of colonies (n = 100) that had more than 5,000 adult bees in the hive on the last day of the year, i.e. we have set the model parameter *CRITICAL_COLONY_SIZE* to 5000. The number of 5,000 bees was conservative, as 4,000 honey bees are often used as an indicator to assess whether bee populations may survive the winter [30]. The simulation continued even if the honeybee population fell below the threshold of 5,000. In such cases, the colony had the option to either recover or go extinct, i.e. the number of honey bees could drop to zero.

#### Interdependencies

**Fig 3:**
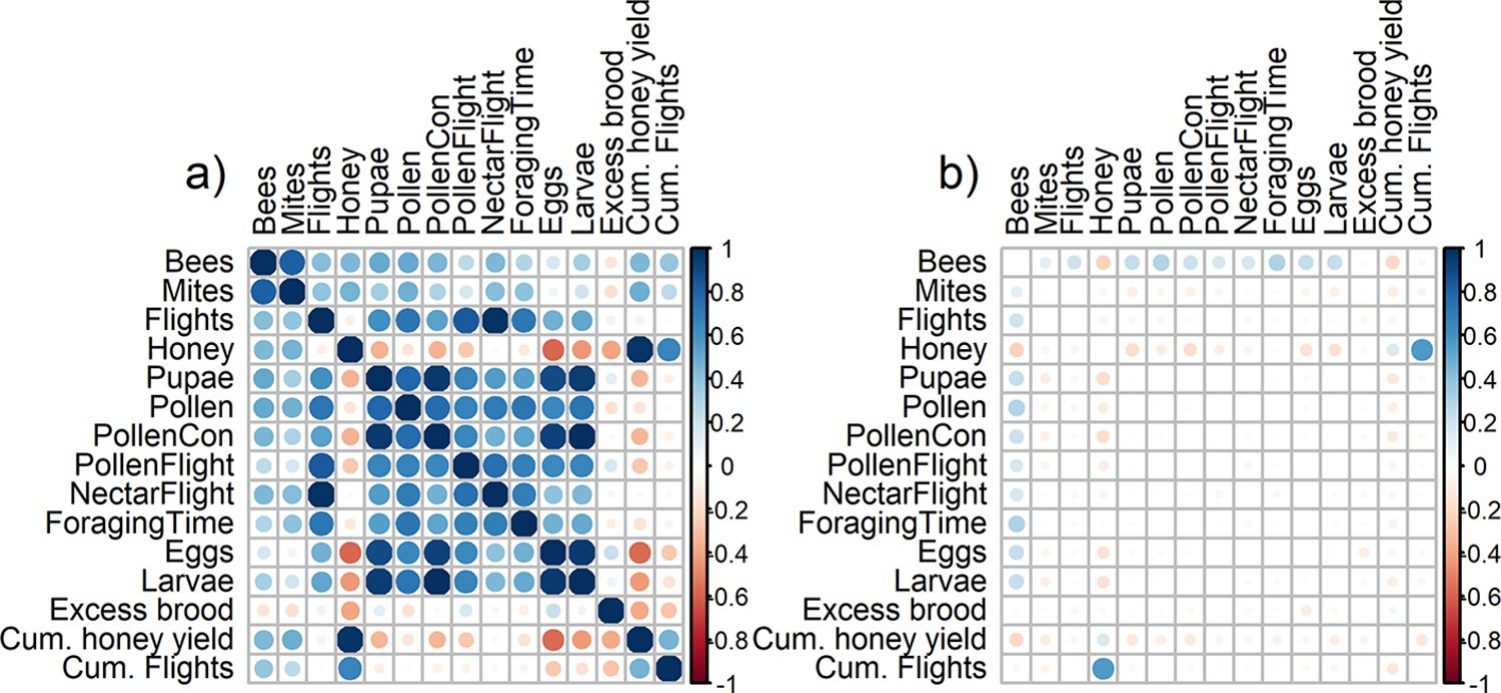
Correlation between outputs of the simulation model for a) the Reference scenario and b) the difference of correlations between the Reference and the Environmental stress scenario. A positive value in the correlation matrix means that the correlation was stronger in the Reference scenario than in the Environmental stress scenario.

In a next step we investigated the temporal correlations between different responses of the simulation model that are potential early warning signals (Fig 3). Therefore, we calculated the Pearson correlation index using the cor() function in R. We used the averages over 100 single runs. The period that was used to compare two time series was between the first day of year 1 and the last day of the second year (before scenarios started to differ in terms of persistence). Thus, each time series contained 730 data points. To demonstrate how resource stress affected the correlation structure of potential early warning signals, we plotted the difference in correlations between the Reference scenario (no stress) and the Environmental stress scenario (Fig 3B). This means that a positive value indicates that the correlation in the Reference scenario was stronger than in the Environmental stress scenario.

#### Identifying indices useful as early warning signals

From 13 simulated responses we selected five responses for more detailed investigation for their usefulness as early warnings signals. These five candidates were selected because they are indicators often measured in experiments. Eggs and larvae were not considered since they are strongly correlated with the number of pupae. We also decided to add the brood-bee ratio as a compound index to the analysis. To quantify the difference between scenarios we measured the difference of the output means (mean of 100 runs). To make these differences comparable between response variables, we divided the difference by the range of this response variable of the Reference scenario and called the computed measure the reduction of a certain output variable *R*, e.g. the reduction of the response variable adult bees *N* for a particular time step t *R*_bee_(t) would be calculated as:

$$R_{bee}(t)=\frac{abs({\hat{N}}_{ref}(t)-{\hat{N}}_{Env}(t))}{\max\left(N_{ref}\right)-min(N_{ref})}$$

Where $\hat{N}(t)$ is the average number (averaged over 100 simulation runs) of adult bees at time *t*. The subscripts of $\hat{N}(t)$ refer to the scenario. max(*N_ref_*) refers to the maximum average number of adult bees over the observation period, e.g., the maximum average number of adult bees in a particular year and min(*N_ref_*) the minimum number, respectively. To assess whether a difference between the Reference scenario and a stress scenario can be realistically detected, we tested whether or not the 90% confidence intervals of the scenarios overlapped. This allowed us to determine the day in the year when the reduction *R* was greater than 10% for the first time and the confidence intervals did not overlap. We call this day *T*_discr_ (*discr* stands for discriminate, i.e., the time when the two scenarios could be discriminated). We also reported the maximum reduction *R*_max_ and the day when this maximum reduction was observed *T*_max_.

#### Testing

Simulation results and thus our conclusions depend on the model parameterization and model settings. Therefore, we tested if and how our results change if we modify details of the implementation of the environmental stress scenario. Hence, we assigned a specific number of stress days per year (*N* = 5, 25, 45, 65, 85, 105, 125, 145, 365) where bees could not forage and measured whether we could distinguish the Reference scenario from the Environmental stress scenario. In the final analysis we replaced time with cumulative foraging time as the independent variable. We only show the results for the early warning index (pupae) that performed well (Table 1) and may be easier to measure than the brood-bee ratio.

**Table 1:** Overview if and when the averages of 100 simulation runs could be distinguished between the reference and the Environmental stress scenario. Stress was detected earliest for the number of pupae (*T*_disc_ = 86 d) and even earlier for the brood-bee ratio and could not be detected at all for the number of flights and number of mites. We also reported the day during the first year of Environmental stress when the maximum difference between average values occurred *T*_max_ and the maximum normalized reduction *R*_max_ at this day (see text for details).

Response	Adult bees	Pupae	Honey	Flights	Mites	Brood-Bee ratio
First day with 10% reduction *T*_disc_	99	85	179	Not detectable	Not detectable	49
Day of maximum reduction *T*_max_	205	166	277	274	268	92
Maximum reduction *R*	47%	45%	49%	35%	52%	38%

## Results

### Population persistence

In Fig 2 we present the persistence that is determined as percentage of honey bee colonies (n = 100) that had more than 5,000 adult bees at the end of a simulated year for three scenarios (Reference, Biotic stress, Environmental stress). All honey bee colonies had survived after the first year. We observed a strong reduction in persistence in the stress scenarios after the third and fourth years (persistence *P* after four years for the reference scenario *P*_ref_ = 95%, Environmental stress *P*_Env_ = 0%, Biotic stress *P*_Bio_ = 23%).

### Interdependencies

Fig 3 shows the correlations among 15 model simulation outputs. We found high correlations between the number of larvae and the daily pollen consumption (ρ = 0.99), and between pollen consumption and the number of pupae (ρ = 0.96). We also found a high correlation between the total number of daily flights and nectar flights (ρ = 0.988). The correlation between total number of daily flights and pollen flights was lower (ρ = 0.83). The strongest negative correlation was found between the amount of honey stored in the hive and the number of eggs (ρ = -0.58) or the number of eggs and the cumulated honey yield (ρ = -0.56). Overall, honey was negatively correlated with the number of honey bees at all life stages. The correlation between honey reserve and cumulative flights was stronger in the Reference scenario than in the Environmental stress scenario (ρ_diff_ = 0.56). The same was observed for foraging time and the number of adult bees (ρ_diff_ = 0.3), the number of adult bees and the number of eggs (ρ_diff_ = 0.2), the number of bees and pollen (ρ_diff_ = 0.24), and the number of adult bees and larvae (ρ_diff_ = 0.24). On the other hand, stored honey and the number of adult bees were less correlated than in the Environmental stress scenario (ρ_diff_ = -0.22).

### Identifying indices useful as early warning signals

**Fig 4:**
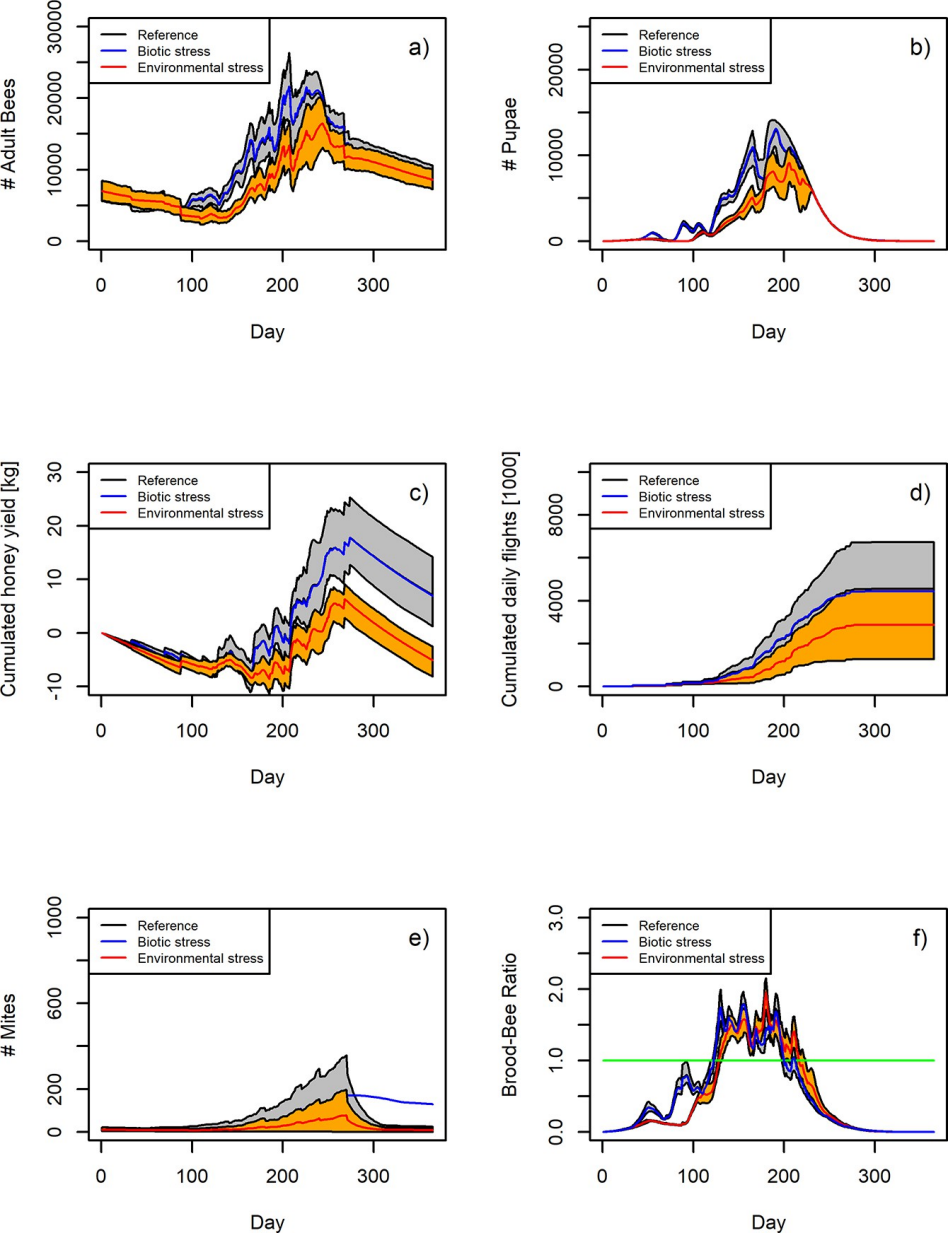
Temporal dynamics of five response variables and the brood-bee ratio for the Reference scenario (black line represents the mean, grey area represents the 90% confidence interval), the Biotic stress scenario (blue line, 90% CI is not shown to increase readability) and the Environmental stress scenario (red line represents the mean and the orange area represents the 90% confidence interval). *N* = 100 runs for all three scenarios. Shown is the first year in which the stress was introduced (after a burn in phase of two years) to assess whether stress can in principle be detected at an early stage.

We contrasted time series of six key model outputs (adult bees, pupae, honey, flights, mites, brood-bee ratio) in Fig 4 during the first year where the bee colonies were exposed to stress. We show the mean of 100 individual runs, accompanied by 90% confidence intervals shown as envelopes. In the appendix, we show the 100 individual time series for each 100 replicates for the number of pupae (S1 Fig in S1 Appendix). For the number of adult bees, the Environmental stress scenario could be distinguished from the reference scenario because the confidence intervals did not overlap (Fig 4A). The earliest day when this discrimination could be detected was *T*_*discr*_ = 99 and the day of maximum discrimination was day 205 with a reduction *R*_*max*_ of 47% (see Fig 4A, Table 1, and section “Identifying indices useful as early warning signals” for further explanation). At the end of the year, it was no longer possible to distinguish between the Reference and Environmental stress scenarios because the confidence intervals completely overlapped. Biotic stress was not measurable at any time using the number of adult bees as an indicator (Fig 4A). For the number of pupae or capped brood cells, the difference between the reference scenario and Environmental stress scenario could be detected earlier (*T*_*discr*_ = 85, *R*_max_ = 45%, *T*_max_ = 166, Fig 4B). Again, the Biotic stress scenario could not be distinguished from the reference scenario by looking at the number of pupae. For the response variable yield index, the averages of the reference scenario and the resource stress scenario could only be distinguished later in the year (*T*_*discr*_ = 179, *R*_max_ = 49%, *T*_max_ = 277, Fig 4C). However, the difference between the Environmental stress and Reference scenarios was evident at the end of the year, in contrast to the demographic response variables of number of adult bees and number of pupae. The model output for cumulative flights (Fig 4D) was not informative because of the overlap of the 90% confidence intervals. For the mite numbers there was a clear signal between both the Reference and Environmental stress scenario compared to the Biotic stress scenario. While for the Reference and Environmental stress scenario numbers of mites dropped towards the end of the year in response to the application of the acaricide, while the Varroa numbers remained high in the Biotic stress scenario (Fig 4E). The brood-bee ratio indicated Environmental stress earliest (*T*_*discr*_ = 49, *R*_max_ = 38%, *T*_max_ = 92, Fig 4F).

### Testing

**Fig 5:**
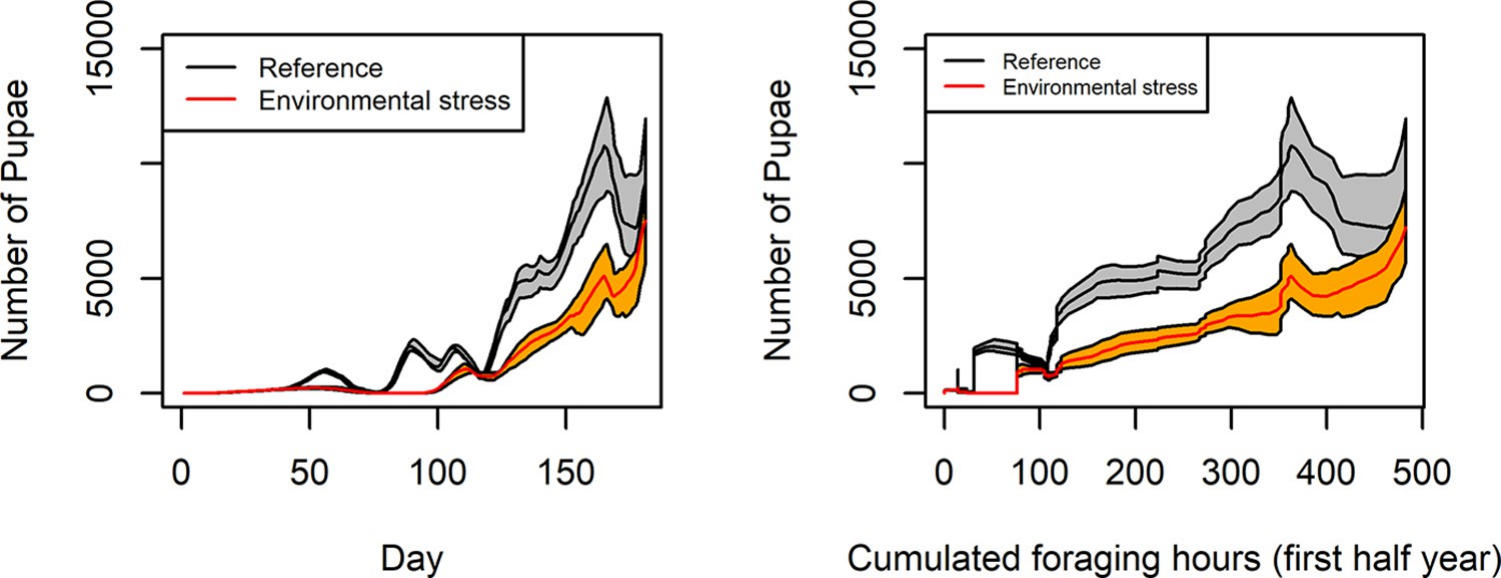
Number of pupae over time and as a function of cumulative foraging hours.

The number of pupae was an early indicator in the year to report Environmental stress and required only one response variable to measure (Table 1). This is why we used pupae as an indicator in the following simulation experiments. Since the flight activity and therefore foraging activity depends on the weather and not only on the time of the year, we tested a weather based independent variable. We used cumulative foraging hours as the independent variable, where foraging hours were defined as daily sunshine hours on days when the maximum temperature exceeded 15°C. The Reference scenario and the Environmental stress scenario showed variation in pupal numbers and total foraging hours over the first 180 days. In particular, the two scenarios could be distinguished after only 50 cumulative foraging hours (Fig 5B).

**Fig 6:**
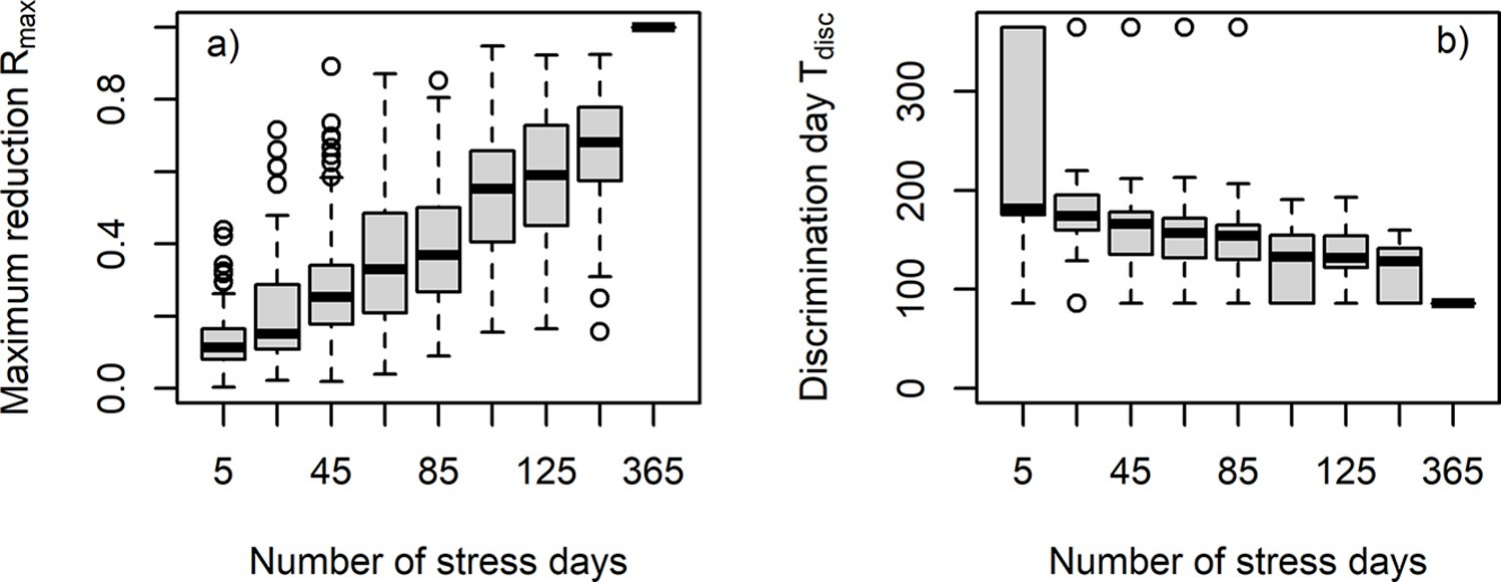
To check the robustness of the results the Environmental stress was applied at random days, i.e. days where the background resource from the grasslands was not available is selected randomly from all 365 days. We varied the number of stress days from 5 to 365 and repeated the simulation 100 times for all numbers. With number of stress days, the maximum reduction of pupae was steadily increasing. The day when the reference scenario could be discriminated from the Environmental stress scenario was declining with the number of stress days.

We also tested the details of the implementation of Environmental stress on the number of pupae in the simulation (see section “Testing” for details). The maximum reduction in the number of pupae in the resource stress scenario compared to the reference scenario *R*_max_ (see section “Identifying indices useful as early warning signals” for definitions) gradually increased with the number of stress days (Fig 6A). The first day at which Environmental stress and Reference scenarios could be distinguished decreased substantially from 5 days of stress per year to 25 days of stress per year. From 25 stress days to 365 stress days the first day when the scenarios could be distinguished decreased only gradually with the number of stress days (Fig 6B).

## Discussion

We used the honey bee simulation model BEEHAVE [30] to investigate which colony-level indicator is suitable to reliably display stress early in the year. Stress was defined as a deviation from the expected development of a bee colony. We tested six indicators: (1) number of adult bees, (2) number of capped brood cells (equivalent to pupae), (3) flight activity, (4) number of Varroa mites, (5) honey reserves, and (6) brood-bee ratio. The simulation experiments were designed in such a way that the imposed stress did not lead to increased winter mortality after the first year. Yet, after three years of exposure, there was a clear strong negative impact, especially in terms of winter losses. Our results indicate that the number of pupae and brood-bee ratio provide reliable information about resource stress in the landscape. However, for biotic stress caused by inadequate Varroa mite control, there is no alternative to Varroa mite monitoring. Monitoring bee health and deriving early warning signals is important for efficient bee management and minimizing bee colony losses [20,21]. Regarding our key finding that monitoring the number of capped brood cells (pupae) is a useful early warning signal, the EFSA Panel on Animal Health and Welfare [21] mentions that based on publications by Collins [41] and Delaplane et al. [42], >10% empty brood cells are a warning of distress. In BEEHAVE the number of potential brood cells is intentionally set to a very high number to avoid an artificial upper limit on the brood size. Thus, we cannot relate directly to the 10% proportion of empty brood cells. The relative index brood-bee ratio is informative, but further research is needed to establish a threshold when the population is at risk. Such a threshold will depend on the day of the year, e.g. in summer a brood-ratio < 1 may indicate stress, while early in the year one would expect a brood-bee ratio < 1 (see Fig 4F, [43]). With a more comprehensive empirical data set, similar to what has already been published for different European beekeeping conditions in spring, summer and fall ([43], see Fig 4 and the following paragraph), a classification system could be developed. This system would make it possible to distinguish between colonies in a normal or critical state, depending on the location. Such a classification could easily be applied to other openly available data sets to test the effectiveness of this indicator.

Other studies reached different conclusions. The modelling study by Hong et al. [44] used critical parameter perturbations and Monte Carlo simulations to examine the effects of multiple factors on colony development. They concluded that monitoring the total number of adults can quickly reflect the health status of a bee colony. In their equation-based model they used three state variables: stock of food, number of adult bees and number of larvae. Capped brood cells (pupae) were not included. In a recent study, Arias-Calluari and colleagues [45] modelled daily weight variation in honey bee hives to develop indicators of bee health. This approach is very interesting especially considering the monitoring networks of automatic scales that measure the weight of bee hives [46,47]. However, our study suggests that yield indices are more meaningful at the end of the season and less useful as early warning signals in the first half of the year. The BEEHAVE model shows structural realism, i.e. effectively replicating a comprehensive set of empirical observations [48,49]. This lends strong support to the idea that examining the number of capped brood cells serves as a valuable and realistic indicator.

Monitoring Varroa mites, even in the absence of clinical signs of disease, is recommended by EFSA as an important health status indicator (expressed as “high link with bee health”) [21,50]. In Europe, Varroa thresholds are typically exceeded in the second half of the year or late in the season, which makes early prediction of mite infestation difficult. In addition, seasonal fluctuations and local conditions also play a role in the mite infestation affecting Varroa thresholds [51]. Therefore, it is recommended to prevent high mite levels with biotechnical measures [40,52]. This was recently considered in BEEHAVE and drone brood removal was implemented [38]. The EFSA report [21] also highlights the importance of assessing honey and pollen reserves at the end of the year. However, we focused here on indicators that inform about problems early in the year highlighting the temporal dimension of bee vitality indicators.

In general, EFSA suggests that for a multidimensional assessment of honey bee colony health, the following elements need to be considered: beekeeping management practices, the resource providing unit with its information on land use and land cover, environmental factors (such as temperature, relative humidity, solar radiation, total precipitation) and five colony attributes (queen presence and performance, in-hive products, diseases, behaviour and physiology, demography) [21]. The BEEHAVE model includes many of these elements, and in this study we focused on four colony attributes as potential indicators. Notably, the presence and performance of queens were not included in our simulations. It is important that these indicators are both meaningful and easily measurable.

The use of simulation models to promote the development of indicators has already been proposed in the field of sustainable forestry [53], but can also be applied to beekeeping to overcome systematic problems: 1) integration between indicators, 2) often studies “do not address the factors giving rise to specific indicator values explicitly” ([53] p.113), and 3) bias toward existing and easily measurable information. Our study highlights the need to consider a number of indicators, since at least the brood, adult bees and the amount of Varroa mites must be considered to characterize the health status of the colony. One possible reason why the number of pupae and brood-bee ratio are the earliest, and therefore most sensitive indicators of Environmental stress is the plausible finding shown in Fig 3A; the number of pupae and pollen consumption, and the number of larvae and pollen consumption are both highly correlated. A lack of pollen directly reduces the amount of brood and, in turn, worker bees. Wang et al. [54] provided empirical evidence that pollen stores were drastically reduced due to paused foraging caused by low temperatures. As a result of this pollen deficiency, brood rearing was severely impaired. This was previously and independently reported by Hellmich and Rothenbuhler [55] and Schmickl and Crailsheim [56], confirming the importance of brood-related indicators.

In general, useful indicators can also be based on changes in correlations between observables. In Fig 3B, we showed how the correlation changes when colonies experienced stress. For example, we demonstrated that in the Environmental stress scenario the correlation between flight activity and stored honey is less pronounced, reflecting the lower honey reserves in the stressed situation. In addition, correlations between different life stages are weakened under stress. In a healthy colony the number of eggs and the number of adult bees would undergo annual cycles with similar amplitudes. In a stressed situation these amplitudes shrink, which can be nicely represented and recognized in an egg/bee phase diagram (see S2 Fig in S1 Appendix). However, establishing reference or expected values for a given colony in a given context and detecting deviations from them will be difficult. One way to make these indices more robust and meaningful might be to use cumulative potential foraging time as the independent variable instead of calendar days (Fig 5B).

In insect modelling, cumulative temperature is often used to describe temporal development [57]. Indicators that are not based on time may be more easily applied between sites. Models are particular useful to extrapolate individual-level responses to stress, such as mortality, to the population level. This is of high relevance since eusocial insects may be able to compensate losses of individuals. Straub et al. [23] argue that bee colonies may be able to buffer the loss of workers as long as breeding is carried on. Such resilience at the colony level has been investigated with BEEHAVE in the context of sublethal effects in response to neonicotinoid application [58]. These simulations suggest that colonies may recover from neonicotinoid application and the resulting sublethal effects, which were still detectable several months after exposure as reduced numbers of adult bees. However, at the end of the year, exposed colonies and control colonies showed the same number of adult bees [58]. If not stressed otherwise, healthy colonies could compensate effects of acute stress at least in the short term.

These results are consistent with our finding that the number of adult bees and other demographic factors such as the number of pupae are useful indicators during the season, but do not provide reliable information at the end of the year. In contrast, honey yield at the end of the year is a reliable indicator of stress, but not so much during the season. It appears that a honey bee colony can compensate for losses in the number of adult bees, but not in resource intake. EFSA considered the threshold of <7% as an acceptable reduction in colony size due to exposure to plant protection products. This "Specific Protection Goal" was introduced when the Bee Guidance Document was first published in 2013 [59]. It is interesting to note that a bee population simulated with BEEHAVE can recover from a 7% reduction in the worker population, suggesting that 20% is more likely to be a critical threshold [60], which is still higher than the recently updated threshold of 10% [61]. This highlights the potential of simulation models to extrapolate measured impacts in time, which is a particularly useful feature for assessing the robustness of indices.

Based on our results, we conclude that the number of pupae and the brood-bee ratio are the most suitable early warning signals. While the brood-bee ratio may be more difficult to monitor because it requires estimates of the number of adults, eggs, pupae, and larvae. The number of pupae can be monitored more easily by estimating the number of broods [62] or by accurate brood photography [54]. However, a prerequisite here is to be able to compare a reference scenario with a stress scenario, and to continuously monitor the number of these pupae. Although we could show that our findings are quite robust towards the temporal regime of the Environmental stress (Fig 6), we appreciate that it will be difficult to compare a monitored scenario and a reference scenario in the field (e.g. number of pupae *vs*. the expected number of pupae in a non-stressed environment). Manual brood estimation is usually performed on a 21-day brood cycle to account for the developmental time of a worker bee [62]. Brood photography is applied within a single brood cycle (i.e., 21 days) and is not designed for continuous monitoring because it is very labour intensive [54]. Thus, future empirical studies are needed to develop an efficient and informative monitoring protocol. Such development would benefit from virtual experiments as presented here.

## Conclusion

Our simulation results suggest that monitoring the number of brood cells and brood-bee ratio are reliable indicators of colony health early in the year. Using the BEEHAVE model, we simulated the development of a single hive without stress and compared this with two stress scenarios to obtain this outcome. The biotic Varroa stress scenario also showed that mite numbers need to be monitored to avoid a cascading effect of a growing Varroa mite population over the years. Therefore, we conclude that the combination of empirical studies and simulation models can be very useful to find meaningful indicators of honey bee vitality. Further research should aim at combining field studies with the need for simulation models and *vice versa* to accelerate progress in this field. This could not only improve the assessment of environmental risks to honey bees, but also provide new opportunities for optimizing colony health in general.

## Supporting information

S1 Appendix S1 Fig. showing annual time series of the brood-bee ratio for 100 individual runs (colonies). S2 Fig. showing a phase diagram of the number of adult bees and number of eggs during two years.
(DOCX)
