# Supplementary material for: Brood indicators are an early warning signal of honey bee colony loss—a simulation-based study
Source: PLoS One. 2024 May 16;19(5):e0302907. doi: 10.1371/journal.pone.0302907 (PMC11098398; doi:10.1371/journal.pone.0302907)
Supplement: S1 Appendix — S2 Fig. showing a phase diagram of the number of adult bees and number of eggs during two years. (DOCX) [file pone.0302907.s001.docx]

Appendix for the manuscript: **Brood indicators are an early warning indicator of honey bee colony loss — a simulation-based study**

**Groeneveld J, Odemer R, Requier F.**


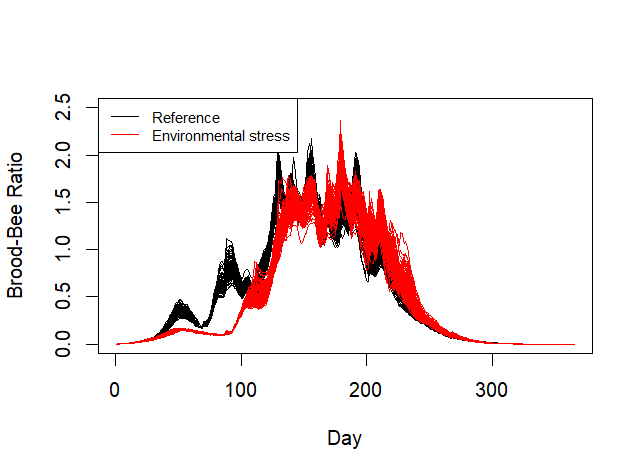


Fig. S1: Annual time series of the brood-bee ratio for 100 individual runs (colonies). During the first 100 days the brood-bee ratio is larger for all colonies compared to colonies exposed to Environmental stress.


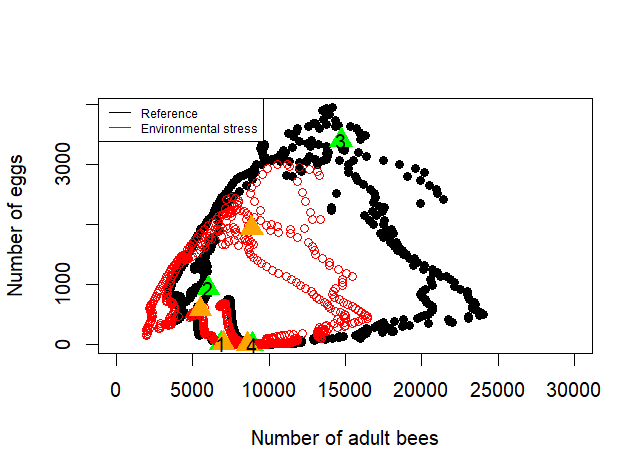


Fig. S2: Phase diagram of two variables: number of adult bees and number of eggs during two years. For a healthy colony all points would lie on a trajectory representing stable annual cycles. In the reference scenario (black points) almost all points are on such a trajectory. The temporal dynamics is illustrated by the green triangles: Starting at point 1 (day 1) going clockwise to point 2 (day 69) , 3 (day 184) and finally 4 (day 365). The stressed colony (red points) has a smaller population size and the cycles will converge towards zero, i.e. the colony will collapse.
